# Cerebellar Hippocampal and Basal Nuclei Transient Edema with Restricted diffusion (CHANTER) Syndrome

**DOI:** 10.1007/s12028-018-00666-4

**Published:** 2019-02-20

**Authors:** Adam S. Jasne, Khalid H. Alsherbini, Matthew S. Smith, Abhi Pandhi, Achala Vagal, Daniel Kanter

**Affiliations:** 1 Yale University, New Haven, CT USA; 2 University of Tennessee Health Science Center, Memphis, TN USA; 3 University of Cincinnati, Cincinnati, OH USA

**Keywords:** Acute brain injuries, Cerebral edema, Drug overdose, Cerebellar syndromes, Hippocampus proper

## Abstract

**Background:**

Abnormal restricted diffusion on magnetic resonance imaging is often associated with ischemic stroke or anoxic injury, but other conditions can present similarly. We present six cases of an unusual but consistent pattern of restricted diffusion in bilateral hippocampi and cerebellar cortices. This pattern of injury is distinct from typical imaging findings in ischemic, anoxic, or toxic injury, suggesting it may represent an under-recognized clinicoradiographic syndrome. Despite initial presentation with stupor or coma in the context of obstructive hydrocephalus, patients may have acceptable outcomes if offered early intervention.

**Methods:**

We identified an ad hoc series of patients at our two institutions between years 2014 and 2017 who presented to the neurocritical care unit with severe, otherwise unexplained cerebellar edema and retrospectively identified several commonalities in history, presentation, and imaging.

**Results:**

Between two institutions, we identified six patients—ages 33–59 years, four male—with similar presentations of decreased level of consciousness in the context of intoxicant exposure, with acute cytotoxic edema of the cerebellar cortex, hippocampi, and aspects of the basal nuclei. All patients presented with severe cerebellar edema which led to obstructive hydrocephalus requiring aggressive medical and/or surgical management. The five patients who survived to discharge demonstrated variable degrees of physical and memory impairment on discharge and at follow-up.

**Conclusions:**

We present findings of a potentially novel syndrome involving a distinct pattern of cerebellar and hippocampal restricted diffusion, with imaging and clinical characteristics distinct from ischemic stroke, hypoxic injury, and known toxidromes and leukoencephalopathies. Given the potential for favorable outcome despite early obstructive hydrocephalus, early identification and treatment of this syndrome are critical.

## Introduction

Cytotoxic edema, identified by restricted diffusion on magnetic resonance imaging (MRI), can be associated with acute ischemic stroke, hypoxic-ischemic encephalopathy (HIE) [1], posterior reversible encephalopathy syndrome (PRES) [2], and various toxidromes [3] including the use of inhaled opiates (i.e., “chasing the dragon”) [4]. When cerebellar and hippocampal edema are seen in HIE from anoxic injury or cardiac arrest, almost invariably other areas of high metabolic demand are also affected, including the cerebral cortex, thalamus, and subcortical white matter [5–7]. Acute obstructive hydrocephalus is not an expected complication of HIE [8]. When patients present unresponsive, with multifocal areas of restricted diffusion accompanied by obstructive hydrocephalus, medical or surgical interventions may not be offered out of the belief that cerebellar edema represents devastating and irreversible cell death, and that patients with these patterns of injury have minimal chance for neurologic improvement despite intervention.

Here, we describe a unique series of cases with a clinicoradiographic syndrome of stupor or coma with imaging findings of bilaterally symmetric abnormally restricted diffusion in the cerebellar cortex, hippocampi, and variable aspects of the basal nuclei. This pattern of acute injury is distinct from previously described conditions and may represent an under-recognized clinical syndrome we term Cerebellar Hippocampal And basal Nuclei Transient Edema with Restricted diffusion (CHANTER) Syndrome. Despite the initial severity of presentation, patients who survive CHANTER syndrome may demonstrate clinical and imaging improvement over time.

## Methods

We identified an ad hoc series of patients seen at the University of Cincinnati Medical Center and University of Tennessee Methodist University Hospital between years 2014 and 2017 who presented to the neurocritical care unit with severe, otherwise unexplained cerebellar edema and retrospectively identified several commonalities in history, presentation, and imaging.

## Results

### Demographics and Clinical Presentation

We identified four patients at the University of Cincinnati Medical Center and two at University of Tennessee Methodist University Hospital with similar imaging findings of severe, bilateral, otherwise unexplained cerebellar cortical edema, identified by restricted diffusion on MRI. In all cases, cerebellar edema was accompanied by restricted diffusion in the bilateral hippocampi and basal nuclei, but sparing the cerebral cortex. Patients ranged in age from 33 to 59 years; two were female and four were male. Five had a prior history of exposure to drugs of abuse and one to prescribed narcotics. Three patients were brought to the emergency department after being found unresponsive in the context of acute opiate exposures; one was brought to the emergency department after using ethanol and stimulants and developing decreased level of consciousness; and one unresponsive after exposures to multiple substances including ethanol, cocaine, and benzodiazepines. One patient received hydromorphone in a post-anesthesia care unit following an elective gastroenterological surgery and became unresponsive and apneic. Patient characteristics, hospital course, and findings are given in Table 1.

**Table 1:** Patient characteristics and outcomes

	Patient 1: 54F	Patient 2: 59M	Patient 3: 49F	Patient 4: 50M	Patient 5: 35M	Patient 6: 33M
Acute exposure	Hydromorphone	BZD, cocaine, opiates	Heroin	Cocaine, fentanyl	Amphetamines, etOH	Cocaine, BZD, etOH
Clinical presentation	Unresponsive, apneic	Unresponsive, hypoxic, extensor posturing	Found unresponsive, cyanotic	Found unresponsive; last well 2 days prior	Encephalopathy, decreased activity	Found unresponsive
Initial GCS	3	5	7	3	11	3
Hospital interventions	MV, HTS, EVD, SubOcc	MV, HTS, EVD, SubOcc	MV, HTS, EVD, SubOcc	MV, HTS, EVD; had preexisting SubOcc	HTS	MV, HTS
Disposition	Trach/PEG, acute rehabilitation	In-hospital death	PEG, shelter home	SNF	Acute rehabilitation	Home with supervision
Follow-up	2 months: moderate cognitive impairment; mild–moderate weakness, wheelchair-bound	n/a	1 month: mild dysarthria only	6 months: cognitive and memory deficits; living at nursing home	3 months: abulia, memory and cognitive issues, mild hemiparesis; living with family	2 years: end-stage renal disease; living alone with home health

*BZD* benzodiazepines, *etOH* ethanol, *EVD* extraventricular drain, *F* female, *GCS* Glasgow Coma Scale, *HTS* hypertonic saline, *M* male, *MV* mechanical ventilation, *PEG* percutaneous endoscopic gastrostomy, *SNF* skilled nursing facility, *SubOcc* suboccipital decompression, *Trach* tracheostomy

### Imaging Findings

**Fig. 1:**
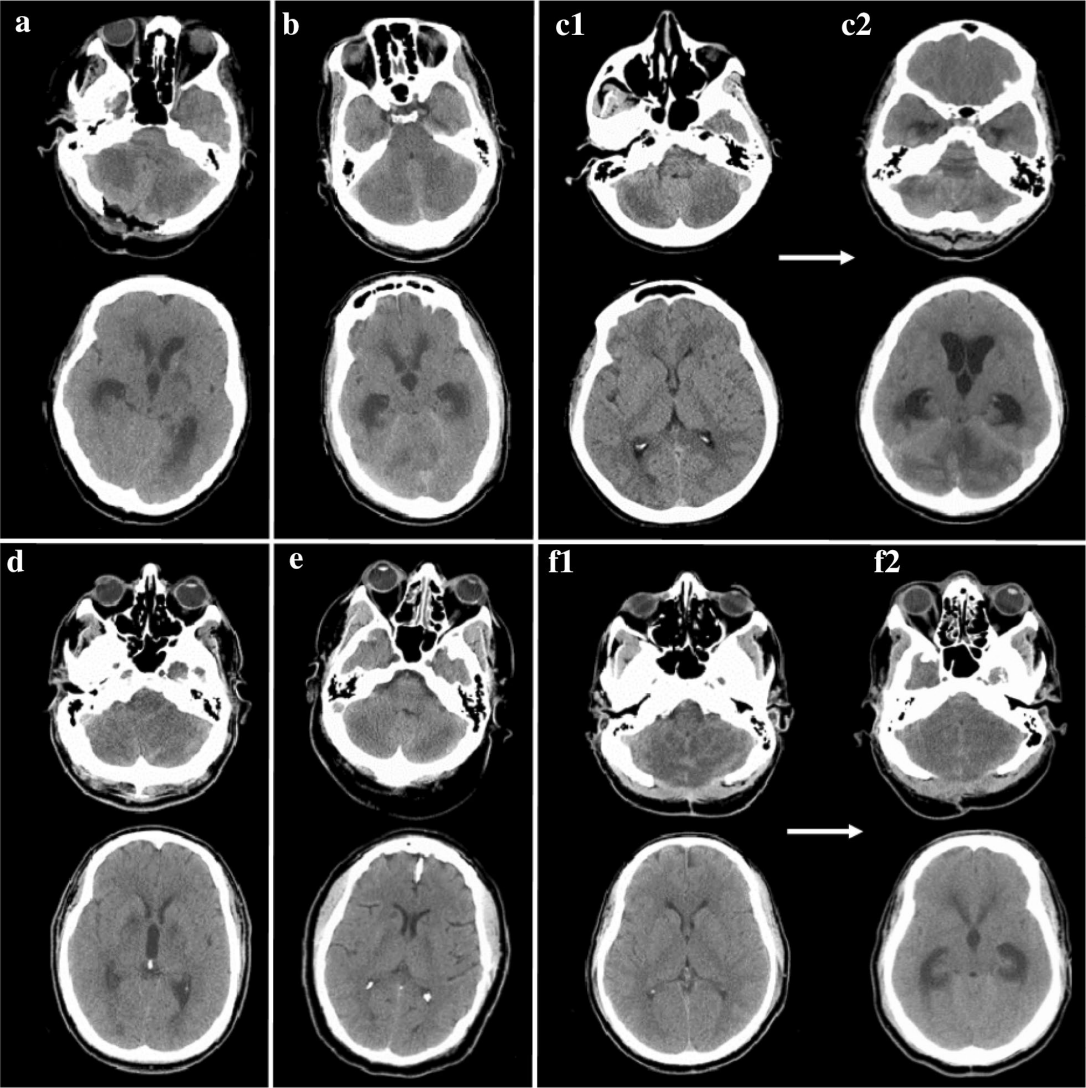
Non-contrast computed tomography of the head from patients at the time of arrival or transfer to tertiary care center, at the level of the cerebellum (above) and the lateral ventricles (below). **A** Patient #1, CT on arrival to tertiary care center, 3 days after initial event; emergent suboccipital decompression was completed on arrival; **B** Patient #2, at time of presentation, ~ 48 h after last well; **C1** Patient #3, on arrival; **C2** Patient #3 on hospital day 6, demonstrating evolution of cerebellar edema with obstructive hydrocephalus; **D** Patient #4, at time of presentation, ~ 24 h from last well; **E** Patient #5, at time of presentation, ~ 24 h from last well; **F1** Patient #4, at time of presentation, ~ 48 h from last well; suboccipital decompression is from a previous event; **F2** Patient #4 on hospital day 4, demonstrating evolution of cerebellar edema with obstructive hydrocephalus

**Fig. 2:**
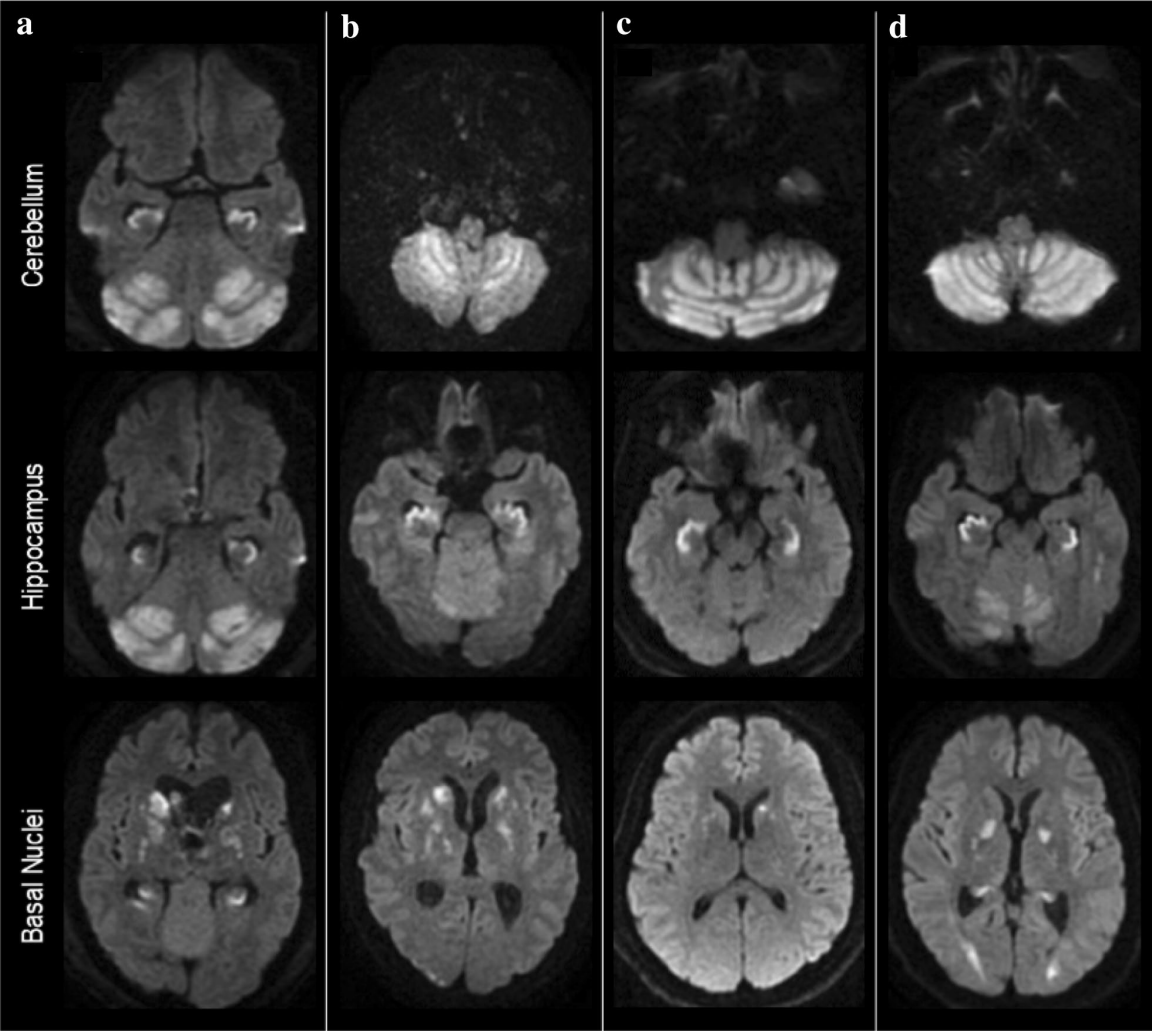
Diffusion-weighted magnetic resonance imaging by patient and brain region demonstrating cytotoxic edema in the cerebellar cortices, hippocampi, and basal ganglia of patients #1 (**b**), #2 (**a**), #4 (**c**), and #5 (**d**). MRI of patients #3 and #6 (not pictured) showed similar patterns

All patients were identified as having acute cerebellar edema on initial evaluation, with progressive worsening. Obstructive hydrocephalus developed in all patients between 0 and 6 days from presentation. Computed tomography at all patients’ time of presentation to tertiary care center is shown in Fig. 1. MRI was performed in all patients and revealed true diffusion restriction (diffusion-weighted imaging hyperintensity corresponding to apparent diffusion coefficient hypointensity) in a bilaterally symmetric distribution in the gray matter of the cerebellar cortex and hippocampi, and asymmetrically in the basal nuclei, sparing the cerebral cortex (Fig. 2). MRI of patient #5 demonstrated additional areas of restricted diffusion in the posterior cerebral white matter; however, no other patients had subcortical white matter involvement. All patients underwent evaluation for other potential etiologies, including electroencephalography and vessel imaging, which did not reveal alternative explanations for the clinical and imaging findings.

### Clinical Course and Outcomes

All patients were admitted to a neurocritical care unit, and all received osmotic therapy with hypertonic saline and/or mannitol. Five of six patients required intubation and mechanical ventilation. Three patients required extraventricular drains for cerebrospinal fluid (CSF) flow diversion and subsequently underwent suboccipital decompressive craniectomy. One patient had suffered an insult 3 years prior, initially diagnosed as a cerebellar ischemic stroke, which was associated with disproportionate cerebellar edema causing obstructive hydrocephalus which required suboccipital decompression at that time. During the hospitalization described in the current series, he required extraventricular drain placement.

**Fig. 3:**
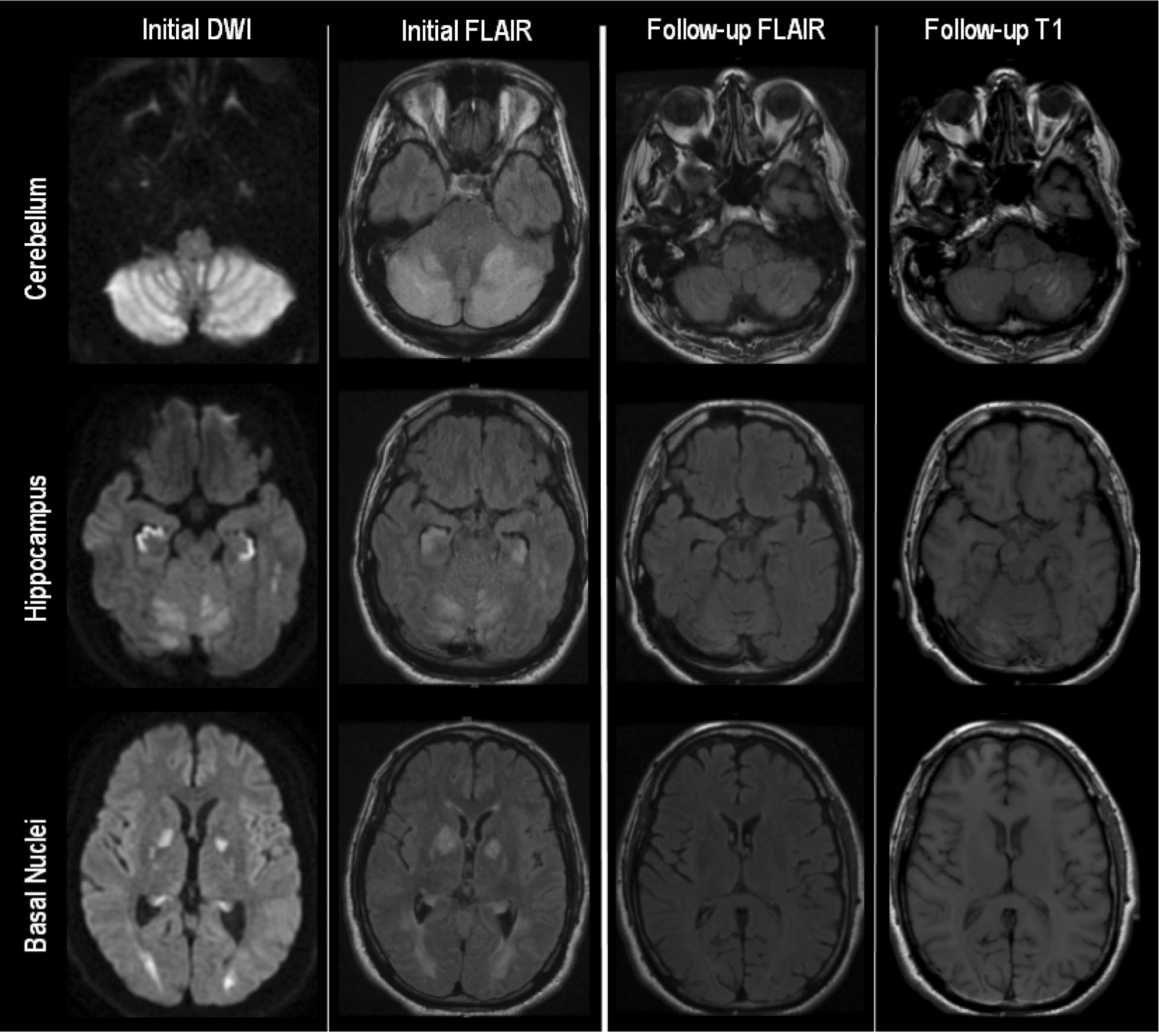
Patient #5 MRI brain, selected sequences, initial and three-month follow-up. *DWI* diffusion-weighted imaging, *FLAIR* fluid-attenuation inversion recovery, *MRI* magnetic resonance imaging. Evolution of imaging changes between initial presentation and repeat imaging 3 months later. Abnormal restricted diffusion was not present on follow-up imaging (not shown)

One patient died during the hospitalization following brain herniation despite aggressive medical and surgical management. Two patients improved to the point of being discharged to home, two were discharged to acute care rehabilitation, and one to a nursing facility. At follow-up, ranging from 1 month to 2 years after the index event, two patients were functionally independent, two lived at home with assistance, and one required care in a skilled nursing facility, primarily due to profound antegrade amnesia. Repeat MRI was available on patient #4 (9 months after index event) and patient #5 (3 months); both demonstrated resolution of restricted diffusion with marked improvement in T2 hyperintensities, with areas of cerebellar laminar necrosis and hippocampal atrophy (Fig. 3).

## Discussion

All patients described in this series presented after acute exposure to opiates or other drugs of abuse and presented with decreased level of consciousness, with obstructive hydrocephalus secondary to cerebellar edema and with MRI findings of restricted diffusion in the cerebellar cortex, hippocampi, and basal nuclei. With aggressive medical and surgical management, most patients were able to live at home with variable degrees of assistance; this may be considered a favorable outcome given the life-threatening nature of the initial injury.

We propose that this constellation of findings represents a condition we term CHANTER syndrome. CHANTER is distinct from other recognized syndromes including acute ischemic stroke, anoxic brain injury, PRES, and described toxic and metabolic injuries. CHANTER shares some clinical and radiographic features with recently described cases of hippocampal restricted diffusion [9–11] and may represent a more severe spectrum of a similar condition.

It is important to distinguish CHANTER from other known clinical entities. Restricted diffusion in CHANTER syndrome may be mistaken for ischemic stroke, but the CHANTER syndrome pattern is not associated with a vascular distribution and does not correspond to vessel occlusions on angiographic imaging.

Heroin-associated spongiform leukoencephalopathy (HASL) is a toxidrome associated with inhaling heroin vapors in a manner termed “chasing the dragon.” It is characterized primarily by extensive, confluent white matter degeneration in the cerebrum and cerebellum [4, 12], in contrast to the gray matter involvement seen in CHANTER syndrome. It is typically described as a subacute process [12] and may not be associated with restricted diffusion [13]. We believe the acute onset of prominent cerebellar and hippocampal restricted diffusion, in the absence of significant white matter injury, distinguishes CHANTER from HASL.

Similar to HASL, PRES is an entity typically associated with predominantly white matter injury. While PRES can cause posterior fossa edema [2], and occasionally involves the basal nuclei [14], it primarily consists of white matter disease which is essentially absent in CHANTER syndrome.

Specific toxic or metabolic insults could produce gray matter injury, but all have significant imaging and clinical differences than CHANTER syndrome, as given in Table 2 [15–20]. One case report describes a patient exposed to carbon monoxide and benzodiazepines who developed injury in a pattern similar to CHANTER [21].

**Table 2:** Distinctive features of CHANTER syndrome, selected differential diagnoses, and similar cases

Etiology	Clinical presentation	Injury/imaging pattern	Other notes
*Syndromes*			
CHANTER	Acute ↓LOC	Bilateral cbel + hippocampi +/− BN	Risk of obstructive HCP
Acute ischemic stroke	Focal neurologic deficits	DWI+ in a vascular distribution	+/− Evidence of vascular occlusion
HASL (“chasing the dragon”) [4, 12, 13]	Strength or movement abnormalities, ataxia; frequently subacute	Predominantly white matter; unlikely DWI+	
PRES [2, 14]	Variable; +/− headache, vision changes, AMS, seizure	Predominantly white matter	Specific provoking factors
Anoxic injury [8, 23–25]	↓LOC	Cerebral cortex +/− cbel, hippocampi, BN	Not typically associated with obstructive HCP
Carbon monoxide (CO) [15, 16, 20, 21]	Headache, AMS	Globus pallidus + BN > cbel + brainstem	Clinical exposure
Cyanide (CN) [17]	Headache, agitation, seizures	BN +/− hippocampi; cbel spared	Clinical exposure
Mercury (Hg) [18]	Acute: systemic symptoms; chronic: personality changes, erethism	Punctate lesions or degeneration without acute edema or DWI+	Clinical exposure
*Similar cases*			
Small/Barash et al. [9, 10]	Memory impairment	Hippocampal DWI+	
Bhattacharyya et al. [11]	Various	Hippocampal and other DWI+ areas	
Pediatric opiate overdoses [26–31]	↓LOC	+/− Cerebellar edema	Limited examples with MRI to show potential other areas of injury

*AMS* altered mental status, *BN* basal nuclei, *cbel* cerebellum, *CHANTER* Cerebellar Hippocampal And basal Nuclei Transient Edema with Restricted diffusion, *DWI+* hyperintensity on diffusion-weighted imaging, *HASL* heroin-associated spongiform leukoencephalopathy, *HCP* hydrocephalus, *LOC* level of consciousness, *PRES* posterior reversible encephalopathy syndrome, *+/−* with or without, *>* more frequently than

Anoxic brain injury following cardiac or respiratory arrest can cause restricted diffusion within gray matter of the hippocampus and cerebellum, specifically within Purkinje cells [22], but this is typically seen co-occurring with cortical gray matter diffusion restriction [23, 24]. Diffuse anoxic injury can be associated with diffuse cerebral edema, but is not typically associated with malignant cerebellar edema and obstructive hydrocephalus [8, 25]. If CHANTER could be considered a variant form of HIE, the disproportionate cerebellar edema with sparing of the cerebral cortex is notable.

CHANTER syndrome may represent a primary metabolic or mitochondrial failure of gray matter with some degree of anoxic injury precipitated by opiates or other drugs of abuse. Single case reports have described cerebellar edema after exposure to opiates, particularly in the pediatric population, either in isolation [26, 27] or with hippocampal [28] or other patterns of injury [29–31]. There are adult case reports of opiate exposure associated with cerebellar edema and hippocampal [32] or basal nuclei damage [33, 34] with some similarities to the cases presented here, although two of these adult cases involved heroin inhalation and were thought to represent HASL-like toxic leukoencephalopathies.

Individuals with active or historical opiate abuse who developed bilateral hippocampal diffusion restriction have been identified, including some with basal nuclei involvement [9]. Recognition of this pattern of injury prompted an alert by the Massachusetts Centers for Disease Control, leading to the identification of ten additional patients with similar findings [10]. The key difference from our case series is the absence of prominent cerebellar edema. A separate case series described sixteen patients with bilateral hippocampal restricted diffusion, including half occurring in the context of recreational drug exposures (most commonly narcotics), and half presumed related to cardiac arrest, seizure, or other etiologies [11]. A majority of patients in this series demonstrated extrahippocampal areas of restricted diffusion, frequently involving the basal nuclei and/or cerebellum. These cases, in conjunction with our own, may suggest the presence of a common pathway and a spectrum of disease severity, with the life-threatening cerebellar edema of CHANTER syndrome representing the most severe end, and isolated hippocampal injury a milder presentation of the same condition. In our series, imaging characteristics and the timing of edema progression are consistent with a pattern of cytotoxic edema, which can explain the development of obstructive hydrocephalus.

Similarities between CHANTER syndrome and these cases raise the possibility of CHANTER as an opiate-provoked toxidrome [35], perhaps exacerbated in the context of relative hypoxia [36]. This concept is indirectly supported by pathologic data. Postmortem analyses of heroin users have identified hippocampal Purkinje cell loss and evidence of reactive micro- or astroglial reactivity believed to be related to a combination of hypoxic and neurotoxic effects [37]. Fentanyl and related synthetic opiates have been linked to focal brain injury, including in the hippocampus [38], suggesting a potential pathophysiologic association. It is possible that interventions such as naloxone—received by patients described individually in the literature [26, 28–32, 34] as well as our cases—offer relative neuroprotection to the cerebral cortex and white matter [39], areas classically implicated in anoxic injury but relatively spared in CHANTER and similar cases. If indeed an opiate-associated phenomenon, the identification of CHANTER syndrome has important clinical implications in light of the ongoing opiate epidemic [40, 41].

While this case series reflects a consistent pattern of clinical and radiographic findings, it shares limitations inherent to all case series. Individuals came to our attention due to otherwise unexplained cerebellar edema resulting evaluation and management in a neurocritical care unit; it is likely that similar cases may not have been identified by virtue of triage to other intensive care units and/or early death due to herniation. While substance use, in particular with opiates, appears to be a commonality between a majority of our patients, and between our patients and those described in the literature, the documented acute exposure in our cases is heterogeneous. This may indicate a variety of provoking factors for a similar syndrome, or it may reflect an underdiagnosis of exposures, such as synthetic opiates that are not a part of standard toxicology assays [42]. An additional limitation is that we do not have histopathological confirmation of the mechanism and precise location of cellular injury. Nonetheless, the strikingly similar clinicoradiographic presentations in our case series suggest a common pathophysiologic process, and the potential need for urgent intervention highlights the importance of recognizing CHANTER syndrome as a potential novel clinicopathologic entity.

## Conclusions

We describe a case series with a clinicoradiographic syndrome of acute, life-threatening cerebellar edema causing obstructive hydrocephalus, possibly provoked by opiates or other drugs of abuse, and associated with symmetric restricted diffusion limited to gray matter of the cerebellum, hippocampus, and basal nuclei. With aggressive medical and/or surgical management of hydrocephalus, some patients are able to achieve reasonable functional outcomes. It is important to differentiate this entity from other disease processes, including ischemic stroke, HASL, or anoxic brain injury. Further work is needed to better understand this syndrome, its risk factors, treatment, and outcomes.
